# Humic substances from composted fennel residues control the inflammation induced by *Helicobacter pylori* infection in AGS cells

**DOI:** 10.1371/journal.pone.0281631

**Published:** 2023-03-09

**Authors:** Mariavittoria Verrillo, Paola Cuomo, Angela Michela Immacolata Montone, Davide Savy, Riccardo Spaccini, Rosanna Capparelli, Alessandro Piccolo

**Affiliations:** 1 Centro Interdipartimentale di Ricerca per la Risonanza Magnetica Nucleare per l’Ambiente, l’Agroalimentare, ed i Nuovi Materiali (CERMANU), University of Naples “Federico II”, Portici, Italy; 2 Department of Agricultural Sciences, University of Naples “Federico II”, Portici, Italy; 3 Department of Food Inspection, Istituto Zooprofilattico Sperimentale del Mezzogiorno, Portici, Italy; University of Brescia: Universita degli Studi di Brescia, ITALY

## Abstract

*Helicobacter pylori* (*H*. *pylori*) is a common human pathogen causing inflammation. Recent studies have suggested a sophisticated interplay between mitochondria, innate immunity and inflammatory response, thus proposing mitochondrial disfunction as the hallmark of severe inflammatory disorders. In this study, humic substances isolated from composted fennel residues (HS-FEN) were tested as potential therapeutical strategy to restore the mitochondrial physiology and control the inflammation associated with *H*. *pylori* infection. The molecular features of HS-FEN were characterized by infrared spectrometry, thermochemolysis-GC/MS, NMR spectroscopy, and high-performance size-exclusion chromatography (HPSEC), which revealed the presence of aromatic polyphenolic components arranged in a rather stable conformation. *In vitro* results showed antioxidant and anti-inflammatory properties of HS-FEN, that was found to increase the expression level of *OPA-1* and *SOD-2* genes and in AGS cells stimulated with *H*. *pylori* culture filtrate (Hpcf) and concomitantly decrease the expression level of *Drp-1* gene and IL-12, IL-17 and G-CSF proteins. The hydrophobic features of HS, their conformational arrangement and large content of bioactive molecules may explain the beneficial effects of HS-FEN, that may potentially become an interesting source of anti-inflammatory agents capable to counteract or prevent the *H*. *pylori*-related inflammatory disorders.

## Introduction

Composting is a highly ecological biotechnology for the management of organic bio-wastes through a controlled microbial process based on an initial intensive degradative thermophilic active step, followed by a mesophilic phase with slow biochemical modification combined with a final stabilization of the composted materials [1,2]. Horticultural vegetables produce a significant amount of crop residues, wastes of agro-industrial transformations and additional unmarketable products that represent an important potential source of organic matter to be potentially reused as compost, soil amendment and fertilizer (Regulation (EU) 2019/1009) [3,4]. Recycling of such residues within farms (On-farm composting) is increasingly adopted as an economic and efficient biological method to re-employ agricultural biomasses [5]. For example, the production of fennel, a common horticultural crop, has grown consistently around the world and Italy is one of the major European producers, with approximately 532,000 tons.y^-1^ [6]. This significant amount of fennel residues contains numerous bioactive compounds, including polyphenols, that may be effectively reused in the medical or nutraceutical fields, thus increasing the added values of these wastes [7,8]. The bioactivity of fennel components is even enhanced after the aerobic microbial transformation during the composting process, and their isolation as humic matter from such a green compost provides an ecological material to be usefully and profitably employed in other remunerative sectors [9].

In this respect, increasing attention is devoted to the development of innovative products to reduce and mitigate the consequences of inflammatory processes in human diseases [10–12]. Inflammation is a defensive response of the host against endogenous or exogenous stimuli, which results in the elimination of harmful signals and return to homeostasis [13]. Inflammation, as well as the oxidative burst, are essential to contain bacterial infections, by removing the invading pathogen and favoring the host healing [14]. However, an unsuccessful host defence, likely due to bacterial or host factors, may lead to a long-lasting inflammatory process, which can cause tissue injury and, therefore, more severe pathological conditions.

*H*. *pylori* is a persistent bacterium, particularly able to evade host defence strategies and promote chronic infection and inflammation. The virulence of the pathogen is directly related to its capability to produce toxins, as well as the different modalities to infect the host [15]. The Vacuolating cytotoxin A (VacA) is the major virulence factor contributing to *H*. *pylori* infection. Through VacA, *H*. *pylori* may affect mitochondrial functions, contributing to the pathogenicity of the infection and the related diseases by promoting inflammation, oxidative stress and cell apoptosis [16–18]. *H*. *pylori* is recognized to be the major cause of gastric diseases and gastric cancer. However, it may also interfere with physiological processes outside the stomach, by inducing a persistent low-grade inflammatory state. Recent studies have reported a strict association between *H*. *pylori* infection and neurological or cardiovascular disorders. In addition, the gut microbiome alteration, closely associated with the *H*. *pylori*-induced inflammation and the massive use of antibiotics to eradicate this microorganism, may promote metabolic disorders such as obesity or diabetes [19].

The failure of common antimicrobial therapies makes *H*. *pylori* infection and the associated- inflammatory diseases a global emergence. In this context, natural organic derivatives, such as humic substances (HS) could provide a significant pharmacological contribute. Based on their heterogeneous molecular composition and flexible conformational properties, HS are recognized to possess interesting antioxidant, antimicrobial, and anti-inflammatory properties [20–23]. The recent advances in their applications in medical therapies make the HS a potential alternative approach to control the *H*. *pylori*-associated inflammation. Furthermore, HS from green composts have also been recently applied as starting material in the synthesis of specialized industrial products or innovative nanomaterials and hydrogels [24–29].

In the present study, we evaluated the antioxidant and anti-inflammatory activity of HS derived from composted fennel residues (HS-FEN), and explored its role in reducing the *H*. *pylori*-associated inflammation, following exposure of AGS cells to *H*. *pylori* culture filtrate.

## Materials and methods

### Green compost and extraction of Humic Substances

Green composts (CO) were produced in Experimental Farm of the University of Naples “Federico II” at Castel Volturno (CE), according to the relevant guidelines and regulations, as reported in Savarese et al., 2022 [2]. Briefly, horticultural residues of fennel crop were mixed with coffee husks at 60/38 w/w plus 2% of mature compost as a starter. The vegetable wastes were placed in static piles with bottom-up oxygen fluxes to ensure aerobic transformation. The composting process lasted 100 days, including the thermophilic and mesophilic phases and a final maturation period. To extract humic substances (HS), finely ground compost (100 g) was suspended in 0.1 mol L^−1^ KOH solution and shaken for 24 h. Then, the extract was centrifuged at 7000 rpm for 20 min and filtered through glass-wool. This extraction was repeated twice (1 h agitation step) and the resulting filtrates were combined. Total extracts, containing both humic and fulvic acids, were acidified to pH 7.4 with 6 mol L^−1^ HCl and dialysed (1 kD cut-off Spectrapore membranes) against deionized water until the electrical conductivity was lower than 0.5 dS m^−1^, and freeze-dried for further analysis.

### Infrared and Solid-state ^13^C NMR spectroscopies

Infrared spectra were recorded on a Perkin Elmer 1720-X FT-IR spectrometer (Waltham, MA, USA), equipped with a diffuse reflectance (DRIFT) accessory, by accumulating up to 8 scans with a resolution of 4 cm^-1^. Samples and oven dried KBr powder were pulverized and mixed in an agate mortar right before spectra acquisition [30].

The solid state ^13^C NMR CPMAS spectrum of HS-FEN was obtained by rotating the sample placed in 4 mm zirconium rotors with Kel-F caps inside wide-bore MAS probe mounted on a Bruker AV-300 magnet with the following acquisition parameters: 13,000 Hz of rotor spin rate; 2 s of recycle time; ^1^H-power for CP 92.16 W: ^1^H 90° pulse 2.85 μs; ^13^C power for CP 150, 4 W; 1 ms of contact time; 30 ms of acquisition time; 4000 scans. The Free Induction Decay (FID) was converted by a 4 k zero filling and an exponential filter function with a line broadening of 100 Hz.

For the interpretation of ^13^C-CPMAS-NMR spectra the overall chemical shift range is split into six regions related to the main organic functional groups: 0–45 ppm (aliphatic-C), 45–60 ppm (methoxyl-C and N-alkyl-C), 60–110 ppm (O-alkyl-C), 110–145 ppm (aromatic-C), 145–160 ppm (O-aryl-C), 160–190 ppm (carboxyl-C)(31)^,^(32). The relative contribution of each carbon group was estimated by relating the intensity of the corresponding spectral interval (Aiabs) to the total area (A0-190abs): Ai% = (Aiabs/A0-190abs) × 100, i = 0–45, 45–60, 60–110, 110–145, 145–160, 160–190. (MestreNova 6.2.0 software, Mestre-lab Research, 2010).

In order to highlight the structural features of humic materials, four dimensionless indexes were calculated from the relative abundance of specific components: O-Alkyl ratio A/OA = [(0–45)/(60–110)]; Aromaticity index ARM = [(110–160)/(0–190)]; Hydrophobic index HB/HI = [(0–45) + (110–160)]/(60–110) + (160–190)]; Lignin ratio LigR = [(45–60)/(145–160)] [31,32].

### Off-line pyrolysis TMAH-GC-MS

Off-line pyrolysis TMAH-GC-MS was performed as described by Verrillo et al. (2022) [33]. Briefly, HS-FEN (500 mg) was placed in a quartz boat and dampened with 1 mL of tetramethyl ammonium hydroxide (TMAH) solution (25% in methanol). The mixture was then dried under a stream of nitrogen and the quartz boat was introduced into a Pyrex tubular reactor (50 cm × 3.5 cm i.d.) and heated at 400°C for 30 min in a horizontal furnace (Barnstead Thermolyne). The products released by thermochemolysis were transferred by a helium flow (20 mL min^-1^) into a series of two chloroform (50 ml) traps kept in ice/salt baths [34]. The extracts were concentrated using rotavapor and the residue was resuspended in 1 mL of chloroform in a glass vial for GC-MS analysis. The identification of release compounds was performed with a Perkin- Elmer GC Autosystem XL by using an RTX-5MS WCOT capillary column (Restek, 30 m × 0.25 mm; film thickness, 0.25 μm), coupled to a PE Turbomass-Gold quadrupole mass spectrometer. The chromatographic separation was carried out according to the following program: 60°C (1 min isothermal), rate 7°C min^-1^ to 320°C (10 min isothermal). Helium was applied as carrier gas at 1.60 mL min^-1^, the injector temperature was at 250°C, the split-injection mode had a 30 mL min^-1^ of split flow. Mass spectra were obtained in EI mode (70 eV), scanning in the range 45–650 m/z, with a cycle time of 1 s. Comparison of mass spectra with the NIST library database, previous published spectra and standard was performed for compound identification.

### High performance size exclusion chromatography

As described by Verrillo et al. (2022) [33], the HPSEC system consisted of a Shimadzu LC-10-AD pump equipped with a Rheodyne rotary injector and 100-μL sample loop and a UV/VIS detector (Perkin e Elmer LC295), set at 280 nm. A PolySep™ GFC-P3000 300 X 7.80 mm (Phenomenex, USA) was employed, and it was preceded by a PolySep GFC-P 35 X 7.80 safety guard (Phenomenex, USA) and a 2 mm inlet filter. The elution flow rate was set to 0.6 mL min^-1^, whereas the eluent was made of 0.1 mol L^-1^ NaH_2_PO_4_ solution (buffered at pH 7.0) added with 4.6 mmol L^-1^ NaN_3_. Prior to the chromatographic analyses, both mobile phase and HS solution were filtered through 0.45 μm Millipore filter. Column calibration was carried out by using sodium polystyrene sulfonates of known molecular masses: 123,000; 16,900 and 6780 Da. Furthermore, ferulic acid (194 Da) and catechol (110 Da) were used as low molecular weight standards. HS-FEN was solubilised in the eluent solution at a concentration of 0.6 g L^-1^ and eluted by HPSEC. In order to verify the conformational stability and molecular size distribution of HS-FEN [35], the same humic solutions were then added with glacial acetic acid (AcOH) to lower their pH to 3.5 and injected again into the HPSEC system. The correlation between molar masses (MM) and elution volumes (EV) provided the following equation: log MM = 0.1407 * EV + 6.4077 (R^2^ = 0.996). The Weight Average (Mw) and Number Average (Mn) molecular weights and polydispersity (P) were therefore calculated. A Unipoint Gilson Software was used to record and elaborate the chromatograms, while the calculations of Mw and P were performed by the Origin software (v. 9.1, Originlab).

### Antioxidant activity of humic extract from fennel composted vegetable wastes

Antioxidant activity of HS-FEN was performed by ABTS assay as described elsewhere [2,9,32]. Briefly, ABTS test was performed in a spectrophotometric method based on the oxidation of 2, 20-azinobis-(3-ethylbenzothiazoline-6-sulphonic acid) diammonium salt (ABTS) by potassium persulphate to form a radical cation (ABTS•+). The ABTS reagent was dissolved in distilled water up to a 7mM concentration to obtain the ABTS stock solution. The ABTS radical cation (ABTS•+) was produced by reacting ABTS stock solution with 2.45mM potassium persulfate (final concentration) and allowing the mixture to stand in the dark for 16 h before use. Then, working solution of ABTS•+ was prepared by diluting the 10 mL of radical cation (ABTS•+) solution with 800 mL of water/ethanol (50:50, v/v) mixture with an absorbance between 0.75–0.80 at 734 nm using UV/vis spectrophotometer.

Solutions of humic samples were prepared at three different concentrations (25, 30,50 μg mL^-1^) in ultrapure water. Then, 100 μl of HS at each concentration were hence added to 1.9 ml of ABTS•+ working solution. The mixture was shaken for 2 minutes at dark to promote the reaction between sample and radical solution and the absorbance was measured at 734 nm. The results were expressed as Trolox Equivalent Antioxidant Capacity (TEAC) by means of a linear calibration curve of Trolox (R^2^ = 0.991).

### Cell culture

Human gastric adenocarcinoma cell line AGS (ATCC, Manassas, VA, USA, #crl-1739) was maintained in Dulbecco’s modification of Eagle’s medium, high glucose (DMEM; Microtech, Naples, Italy), supplemented with 10% fetal bovine serum (FBS; Microtech, Naples, Italy), 1% penicillin/streptomycin (Gibco, Waltham, MA, USA) and 1% L-glutamine (Gibco, Waltham, MA, USA) in a humidified atmosphere at 37°C and 5% CO_2_.

### *Helicobacter pylori* culture filtrate preparation

*Helicobacter pylori* culture filtrate (Hpcf) was prepared as described by Cuomo et al. [36]. In detail, culture filtrate of *H*. *pylori* P12 strain was prepared by culturing the bacterium on selective Columbia agar (Oxoid, Basingstoke, Hampshire, UK) supplemented with 7% (v/v) of defibrinated horse serum (Oxoid, Basingstoke, Hampshire, UK) and antibiotic mix (DENT; Oxoid). Bacteria plates were incubated for 3–4 days at 37°C in a 10% CO_2_ atmosphere. After growth, bacteria were scratched using brain heart infusion (BHI; Oxoid, Basingstoke, Hampshire, UK) and measured. 2 x 10^7^ bacteria were cultured in DMEM (Microtech, Naples, Italy) supplemented with 10% FBS (Microtech, Naples, Italy) and incubated at 37°C in a 10% CO_2_ atmosphere for 24 hours. Finally, bacterial suspension was centrifuged at 10,000 g for 10 minutes and the supernatant was filtered by using a 0.22 μm filter.

### Cell viability assay

AGS cells were split at 80–90% of confluence, seeded (2 x 10^4^) in a 96-well plate and incubated at 37°C in a 5% CO_2_ atmosphere overnight. After cell attachment, the medium was replaced with fresh one, serum-free, containing different concentrations of the HS-FEN (500 μg/mL, 250 μg/mL, 100 μg/mL, 50 μg/mL, 25 μg/mL, 12.5 μg/mL, 6 μg/mL) or Hpcf (Hpcf as it is or diluted 1:2, 1:4, 1:8 with uninoculated growth broth) and cells were incubated for 24 hours. After cell washing, medium containing 3–4,5-dimethylthiazol 2,5-diphenyltetrazolium bromide solution (MTT 1:10; Merk, Darmstadt, Germany) was added to each well and cells further incubated at 37°C in a 5% CO_2_ atmosphere for 3 hours. Finally, the medium was removed, and the resultant formazan crystals dissolved in 200 μL of DMSO. Cellular MTT reductase activity was determined by measuring the absorbance of DMSO extracts at 570 nm, using an EnVision 2102 multilabel reader (PerkinElmer, Waltham, MA, USA). Results are expressed as the percentage of MTT-reducing activity of treated vs. untreated cells, according to the following formula:

% cell viability = [(mean absorbance of the sample)—(mean absorbance of the blank)]/[(mean absorbance of the control)— (mean absorbance of the blank)] x 100.

### Multiplex cytokine measurement

AGS cells were split at 80–90% of confluence, seeded (1 x 10^6^) in a 12-well plate and incubated at 37°C in a 5% CO_2_ atmosphere overnight, until cell attachment. Cells were washed and treated with: 1) Hpcf diluted 1:2 with uninoculated growth broth (1:2); 2) Hpcf diluted 1:2 with uninoculated growth broth (1:2) + HS-FEN 25 μg/mL. After 12 hours of treatment, medium was collected and centrifugated at 10,000 × g to remove debris and dead cells and analysed for the concentration of IL-17; IL-2 and G-CSF cytokines, by using the Bioplex Multiplex human cytokine assay (Bio-Rad), as indicated by manufacturer’s instructions. Briefly, 96-well plate was coated with 50 μL of microspheres (magnetic beads) covalently coupled to capture antibodies able to detect specific cytokines (IL-12, IL-17 and G-CSF). After coating, coupled beads were washed two times with 100 μL of wash buffer by vacuum filtration. 50 μL of cytokine standards (at different dilutions), samples or controls were added, and the plate was incubated for 30 minutes at room temperature on a shaker. Each standard was run in duplicate, while samples or controls in triplicate. The plate was further washed three times with 100 μL of wash buffer by vacuum filtration. 25 μL of biotinylated detection antibody were then added to each well, in order to create the sandwich complex, and the plate was incubated for 30 minutes at room temperature on a shaker. Following this second incubation, the plate was washed as before, and 50 μL of streptavidin- phycoerythrin were added to each well. 10-minute following incubation and after final wash, the beads were resuspended in 125 μL of assay buffer. The 96-well plate containing the resuspended microspheres was placed in the Bio-Plex 200 System (Bio-Rad) and data analysed by Bio-Plex Manager^TM^ Software. Identification and quantification of cytokine is determined by the median fluorescence intensity of the fluorescent dye phycoerythrin. Phycoerythrin is excited at 635 and 532 nm to generate a report signal. The median fluorescence intensity of the unknown sample is then converted into concentration (pg/mL) based on the known cytokine concentrations of the standard curve.

### RNA extraction and gene expression measurement

Expression levels of *OPA1*, *SOD2* and *Drp-1* genes were measured by RT-PCR. AGS cells were seeded (2 x 10^6^) in a 6-well plate and incubated at 37°C in a 5% CO_2_ atmosphere overnight, until cell attachment. The next day, cells were incubated with Hpcf (1:2) for 3 hours in presence or not of HS-FEN 25 μg/mL. After incubation, RNA was extracted using TRIzol LS reagent (Thermo Fisher Scientific). Briefly, cells were lysed in 1 mL of Trizol reagent and incubated on ice for 5 minutes. 200 μL of chloroform were added and each sample was centrifuged at 13,000 rpm, 4°C for 15 minutes. The aqueous phase (upper phase) was transferred into a new tube and 500 μL of isopropanol were added. The mixture was then vortexed, incubated on ice for 5 minutes and centrifuged at 13,000 rpm, 4°C for 10 minutes. Supernatant was discarded and RNA pellet suspended using ethanol 75%. After centrifugation (13,000 rpm, 4°C for 7 minutes), supernatant was removed, and pellet eluted in 30 μL of RNase free water. The quality and quantity of RNA was assessed using NanoDrop 2000c (Thermo Fisher Scientific). cDNA was finally prepared using the high-capacity cDNA Reverse transcription kit (Thermo Fisher Scientific).

Real-time PCR reactions were performed on a StepOne Real-Time PCR System (Thermo Fisher Scientific), using Power SYBR Green PCR Master Mix (Applied Biosystem) as amplification system [37]. Gene-specific primers are listed in S2 Table. The 2^- ΔΔCt^ method was used to determinate relative changes in target gene expression. As housekeeping gene, GAPDH was used as internal control, to normalize data.

### Statistical analysis

GraphPad Prism 6.0 (San Diego, CA, USA) was used to analyze data. Multiple comparisons, among more than two experimental groups, were performed using One-way ANOVA followed by Bonferroni *post-hoc* correction. Data were considered statistically significant when *p* value was < 0.05.

## Results and discussion

### Infrared spectroscopy

**Fig 1:**
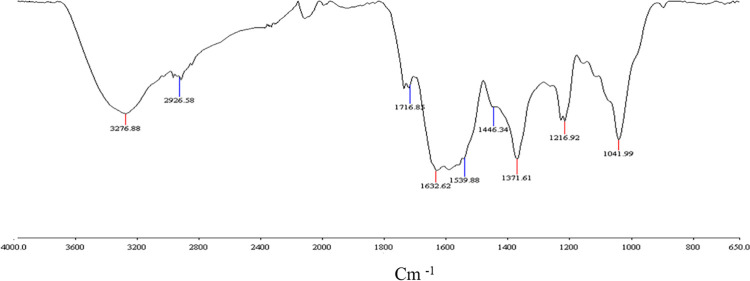
FTIR-DRIFT spectrum of humic substances from fennel green compost.

The DRIFT spectrum of HS-FEN (Fig 1**)** showed a broad absorption band around 3000–3500 cm^−1^ derived from the overlap of the intense OH stretching vibrations in alcohols and phenolic compounds and carboxylic acids. The bands at 2950–2920 cm^−1^ are referred to symmetric and asymmetric C-H stretching of methyl and methylene groups in aliphatic chains of lipid compounds, associated with the weak bending vibration at 1440 cm^-1^ [30]. The inclusion of alkyl carboxylic groups is indicated by the doublet at 1714 cm^-1^ of carboxyls, coupled with the less intense shoulder related with C-O bending at 1220 cm^-1^. The signals at 1620 cm^-1^ and 1540 cm^-1^ may be related to either amide I or amide II bonds of peptides [31], or ring vibrations of aromatic moieties [4,31]. The prominent band at 1367 cm^-1^ is attributed to phenolic functional groups and aryl ether bonds [38]. Finally, the bending of C-O bonds at 1040 cm^-1^ suggests the presence of hydroxyl functions in carbohydrates and polysaccharides (Fig 1).

### NMR spectroscopy

**Fig 2:**
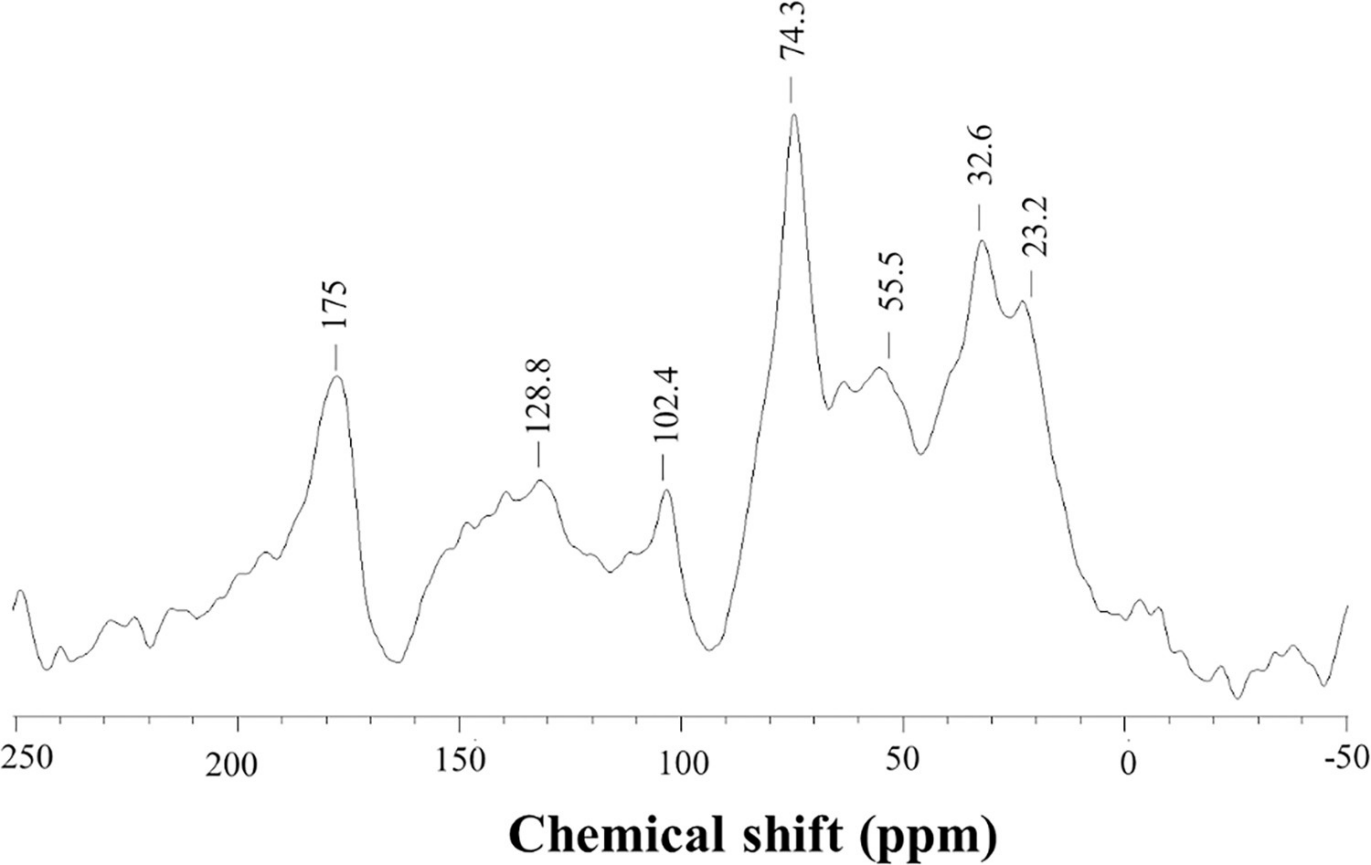
^13^C-CPMAS-NMR spectrum of HS-FEN.

The ^**13**^C-CPMAS NMR spectrum of HS-FEN (Fig 2) confirms not only the presence of both apolar alkyl, aromatic compounds and polar components related to C-O and C-N containing molecules, as already inferred by the IR spectrum, but also the general features proper of HS from aerobic composts [31,39]. The structural features of HS may be also inferred by the dimensionless structural parameters calculated from the relative amounts of carbon distribution over intervals of the spectral range (Table 1). The value of hydrophobic index close to unity, indicates a uniform partition of C between polar and apolar functional groups mainly determined by the aliphatic components as suggested by the similar result found for the alkyl ratio (Table 1). However, the ARM index suggests a significative content of aromatic molecules, while the low level of LigR shows a close correlation of spectral intensities associated to methoxyl groups (45–60 ppm) and O-aryl-C molecules (140–160 ppm), thus revealing the prevalent contribution of lignin fragments and phenolic compounds in these spectral regions (Table 1).

**Table 1:** Relative distribution (%) of signal areas over chemical shift regions (ppm) and structural indexes^a^ in ^13^C CPMAS-NMR spectrum of HS.

Sample	200–160 Carboxyl-C	160–145 O-aryl-C	145–110 Aryl-C	110–60 O-Alkyl-C	60–45 CH_3_O/C-N	45–0 Alkyl-C	A/OA	ARM	HB/HI	LigR
**HS-FEN**	12.4	8.5	12.2	24.9	16.1	25.9	1.0	0.4	0.9	1.9

^**a**^ A/OA = (0–45)/ (60–110); ^**b**^ ARM = [(110–160)/Σ(0–45) + (60–110)]; ^**c**^ HB/HI: Σ[(0–45) + (45–60)/2+(110–160)] / Σ[(45–60)/2+(60–110)+ (160–190)]; ^**d**^ Lig R: (45–60) / (145–160).

### Off-line TMAH-Pyr-GC–MS

The thermochemolysis HS-FEN released methyl ethers and esters of alkyl and aryl compounds of plant and microbial origin, identified as lignin derivatives (Lig), linear fatty acids (FAME), nitrogen-containing (N) compounds, alicyclic lipid (e.g. sterols) and carbohydrates (S1 Fig and S1 Table). The fact that a relatively smaller amount of carbohydrates can be inferred in the thermochemolysis of HS-FEN (Table 2) in respect to the NMR spectrum is due to the poor analytical efficiency of thermochemolysis in detecting polyhydroxy molecules in complex matrices [1]. The most abundant compounds in HS-FEN pyrograms were lignin monomers, which were classified according to the principal structures found in the lignified tissues of higher plants: P = p-hydroxyphenyl, G = guaiacol (3-methoxy, 4-hydroxyphenyl), and S = syringyl (3,5- dimethoxy, 4-hydroxyphenyl) [40]. The most abundant lignin derivatives were the oxidized products of both di- and tri-methoxyphenylpropane, such as benzaldehyde (G4 and S4), acetophenone (G5 and S5), and benzoic acid (G6 and S6). Other identified lignin units were cis and trans isomers of 1-(3,4-dimethoxyphenyl)-2-methoxyethylene (G7 and G8) and 1-(3,4, 5- trimethoxyphenyl)-2-methoxyethylene (S7 and S8), as well as the enantiomers of 1-(3,4-dimethoxyphenyl)-1,2,3-trimethoxypropane (G14 and G15) and the 1-(3,4,5-trimethoxyphenyl)-1,2,3-trimethoxypropane (S14 and S15). The 3,4-dimethoxyphenyl-2-propenoic acid (G18) compounds originated from either lignin guaiacyl units or suberin aromatic domains. Additionally, other aromatic compounds were identified, such as phenols, methyl-phenols, and alkyl-benzenes derivatives, which may have multiple origins (polysaccharides, proteins, lignin, polyphenols). Besides the total distribution, the relative amounts of specific lignin-derived compounds may provide useful indications [1]. Structural indexes informative of the lignin decomposition stage may be calculated by dividing the area of the oxidized 3,4-dimethoxylbenzoic acid and 3,4,5-trimethoxylbenzoic acids (G6 and S6) over the corresponding aldehydic forms (G4 and S4). The larger values found for the two structural parameters (Table 2), suggest that the composting process produces an extensive lignin decomposition into lignin fragments smaller in molecular size [41].

**Table 2:** Relative yield (%) of main thermochemolysis products a released from HS-FEN.

Thermochemolysis products HS-FEN	%
Lignin	59.7
Ad/AlG^a^	6.2
Ad/AlS^b^	14.8
Aromatic (non-lignin)	7.4
N derivatives	18.7
FAME ^c^	10.5
Carbohydrates	0.4
Sterols	2.3
Alcohols	1.9
Alkanes	0.2

^**a**^: Ad/AlG = [G6/G4]; ^**b**^: Ad/AlS = [S6/S4]; ^**c**^: Fame: fatty acid methyl ester.

### High performance size exclusion chromatography (HPSEC) of HS

**Fig 3:**
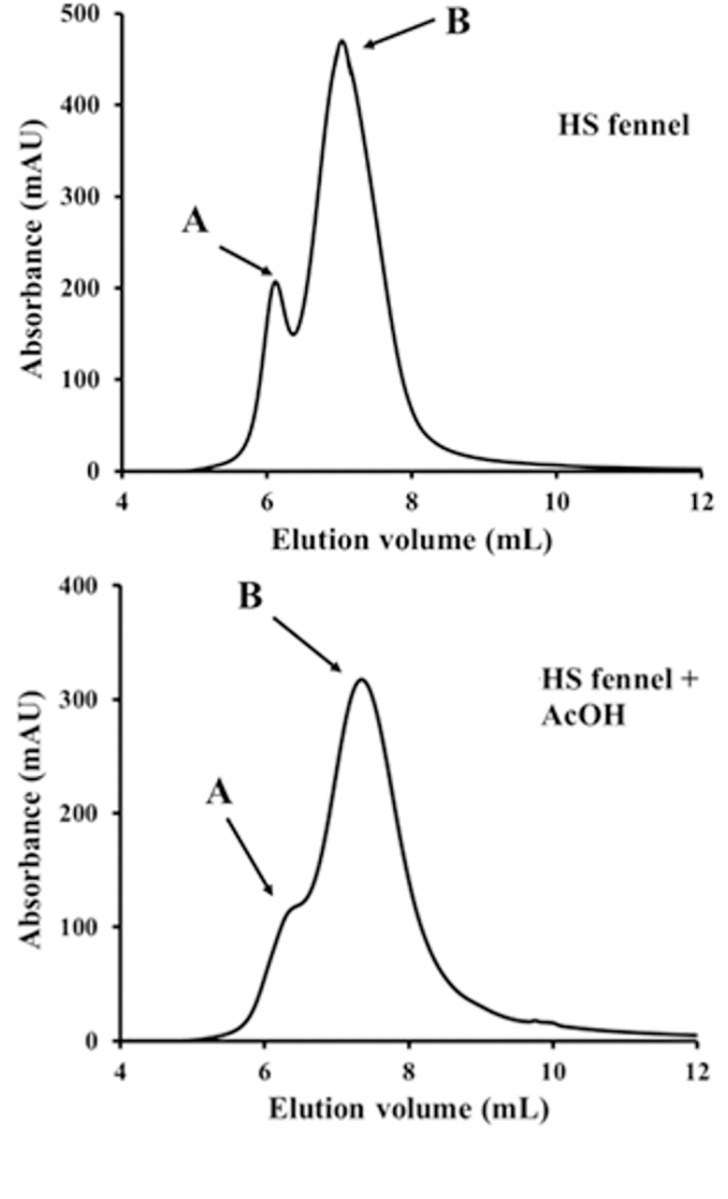
HPSEC chromatograms of HS-FEN before and after addition of acetic acid (AcOH) to lower solution pH from 7 to 3.5.

The HPSEC chromatograms of HS from composted fennel wastes are shown in Fig 3, while the relative nominal weight-averaged (Mw) and number-averaged (Mn) molecular weights are reported in Table 3. This humic material showed a bimodal distribution of molecular sizes, as commonly observed for HS of various origins [33,42]. The nominal Mw and Mn for peak A were 83600 Da and 82400 Da, respectively, whereas they were 45200 and 36600 Da for peak B (Table 3). Due to the similar Mw and Mn values, both peaks had a monodisperse nature, with a polydispersity value equal or very close to 1.0 (Table 3).

**Table 3:** Weight average (Mw) and number average (Mn) molecular weights, and polydispersity (P), as calculated from UV-detected HPSEC chromatograms for HS-FEN, before and after addition of acetic acid (AcOH). Standard deviation was < 5%.

Sample (peak interval-mL)	Mw	Mn	P
HS-A (4.8–6.4)	83593	82413	1.0
HS-B (6.4–13.1)	45170	36578	1.2
HS + AcOH-A (4.8–6.5)	78574	77059	1.0
HS + AcOH-B (4.8–14.1)	37206	23883	1.6

Another HPSEC run was conducted after having treated the humic solution with acetic acid (AcOH) to lower the pH from 7 to 3.5, in order to verify the conformational stability of this humic sample. The addition of AcOH drives the formation of novel hydrogen bonds among complementary oxygen-bearing compounds, which alter the weak interactions stabilising the humic suprastructures at pH 7 and induce a dispersion of the newly smaller formed suprastructures through the HPSEC pore distribution [35,39]. Only a significant decrease in the absorbance of peak A and a slight shift towards larger elution volumes for peak B were observed upon AcOH treatment (Table 3, Fig 3). These changes can be thus explained with a fragmentation of the hydrophobic components of HS which were originally associated into nominally large molecular sizes (peak A) and were then diffused through the column towards larger elution volumes (peak B). The substantial stability of the conformation of HS from the green fennel compost upon variation of the solution pH, suggests that the saccharidic components revealed by the above spectroscopic results, still maintained a macromolecular character and were associated with smaller sized hydrophobic components. Similar results were recently reported for other HS from composted agricultural residues which appeared to contain plant derived lignin and cellulose not yet completely depolymerized after the composting process [2,33,40].

### Antioxidant activity of humic substances by fennel green compost

**Fig 4:**
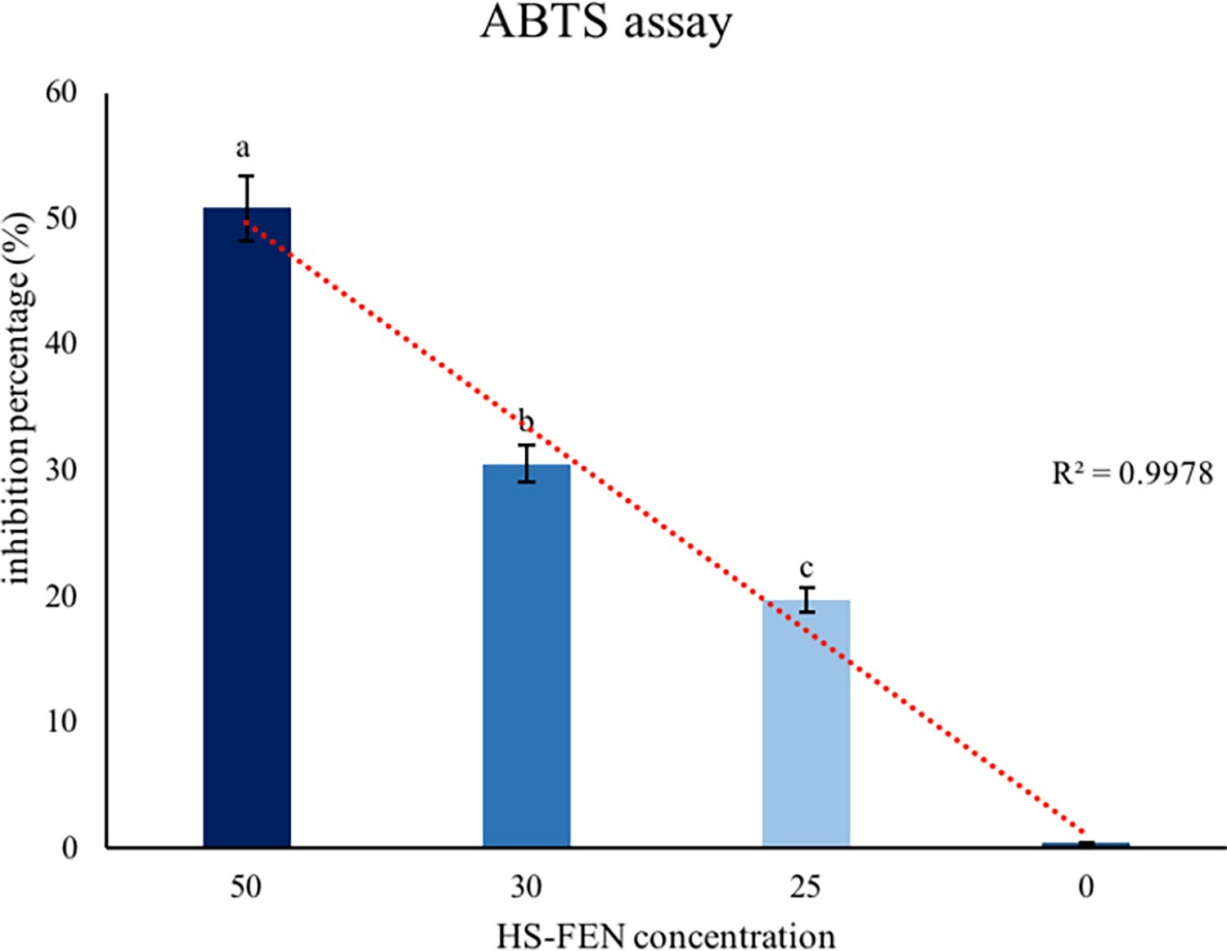
Antioxidant activity of HS-FEN at different concentration (50, 30, 25 μg mL^-1^). Vertical bars represent the standard deviation (s.d.). Different capital letters indicate significant differences according to Tukey test (*p* ≤ 0.05).

Antioxidant substances provide a significant protection against various diseases that are related to oxidative stress generally induced by free radicals, such as reactive nitrogen species (RNS) and reactive oxygen species (ROS) [43]. For this reason, radical scavenger activities of phenolic components in natural molecules with their electron donors/acceptors behavior, have been extensively discussed [44]. Humic substances were recently used for medical application as natural antioxidants since they were shown to efficiently inactivate free radicals due to their abundant content of reducing molecular components [33,40]. Here we found that HS-FEN revealed a significant free radical percent inhibition at the maximum concentration tested (50.86%), though a clear dose effect was also visible (Fig 4). Similar results were obtained by expressing the antioxidant ABTS results against the standard antioxidant Trolox as TEAC (mmol of Trolox equivalent・kg^−1^ of sample), showing values such as 397.5 for HS-FEN at 50 μg mL ^-1^ followed, by 285 and 173 for the lower concentrations of 30 and 25 μg mL ^-1^, respectively. In line with previous studies, these data indicate a clear correlation between the phenolic content of this humic sample (Table 1 and Fig 2) and its antioxidant capacities [4,9,32]. Although antiradical and antioxidant activities of several humic substances have been already discussed [45], no literature information is reported regarding specific HS isolated from composted fennel residues, which may represent an important source of bioactive material for the pharmaceutical industry [46]. Our results, which relate the antioxidant activity of HS-FEN to its content of phenolic moieties (Tables 1 and 2), are in agreement with other studies on phenolic extracts, in which mono- and oligo-hydroxylated benzene units are responsible for the antioxidant properties [47], as major electron donating groups in humic samples [48].

### The effect of HS-FEN on cell viability

**Fig 5:**
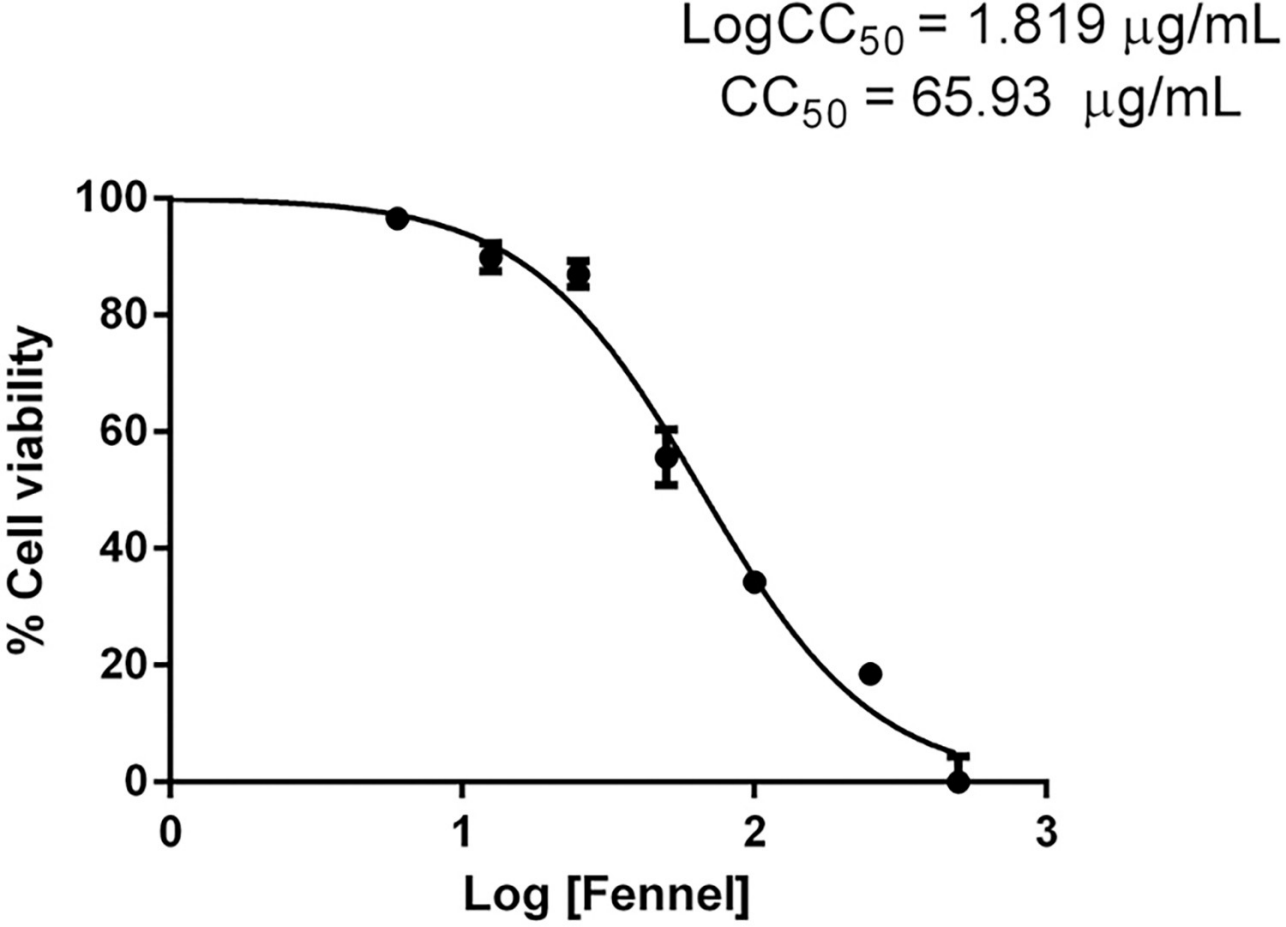
Cell viability and CC_50_. MTT assay showing the AGS cells viability after incubation with increasing concentrations of HS-FEN (spanning from 6 μg mL^-1^ to 500 μg mL^-1^) for 24 hours. The concentration of 65.93 μg/mL was estimated as that inhibiting 50% of cell viability (CC_50_).

The capability of the selected HS (HS-FEN) in affecting the metabolic activity of human gastric adenocarcinoma cell line AGS was assayed by performing the MTT assay. Cells were incubated with different concentrations of HS (from 500 μg mL^-1^ to 6 μg mL^-1^) for 24 hours. As shown in Fig 5, HS-FEN was found not to be toxic at concentrations lower than 50 μg mL^-1^, since cell viability was larger than 80%, as compared to control cells. We also determined the concentration of HS-FEN responsible for 50% decrease of cell viability (CC_50_). Results showed 50% cytotoxic concentration at 65.93 μg mL^-1^. Concentrations of HS-FEN responsible for a significant reduction of cell viability (< 80%) were excluded. This result, again, confirms the large content of lignin fragments in HS-FEN (Tables 1 and 2). High concentrations of lignin, in fact, are known to show cytotoxic effects [49]. It is important to consider that usually the antioxidant activity of molecules is expressed to cytotoxic concentrations. Interestingly, lignins show antioxidant capacity to non-cytotoxic concentrations [49] (Fig 4). Therefore, these data have an important implication on the potential clinical application of HS-FEN, suggesting that the concentration of HS-FEN required for beneficial effects is safe for the analyzed cell line.

### HS-FEN protects AGS cells from the Hpcf-induced oxidative stress and inflammation

*H*. *pylori* is the main environmental agent responsible for chronic gastric inflammation, which may result in chronic gastritis and gastric cancer. Although the mechanism disclosing the relationship between *H*. *pylori* infection and gastric cancer has not yet been elucidated, the imbalance between cell proliferation and apoptosis could represent one of the factors contributing to gastric mucosa damage and malignant transformation [50].

*H*. *pylori* secretes a variety of bacterial toxins and antigens that, upon recognition by gastric epithelial cells, can impair gastric mucosa integrity, inducing cell apoptosis via stimulation of pro-inflammatory mediators [51].

The vacuolating cytotoxin A (VacA) is the major virulence factor produced and released by *H*. *pylori* [52]. VacA can enter different host cells and exert different functions, resulting in alteration of cell physiology [19,52,53]. Inside the cells, VacA localizes in the mitochondria and initiates the mobilization of cytochrome c, leading to apoptosis and oxidative stress [54].

**Fig 6:**
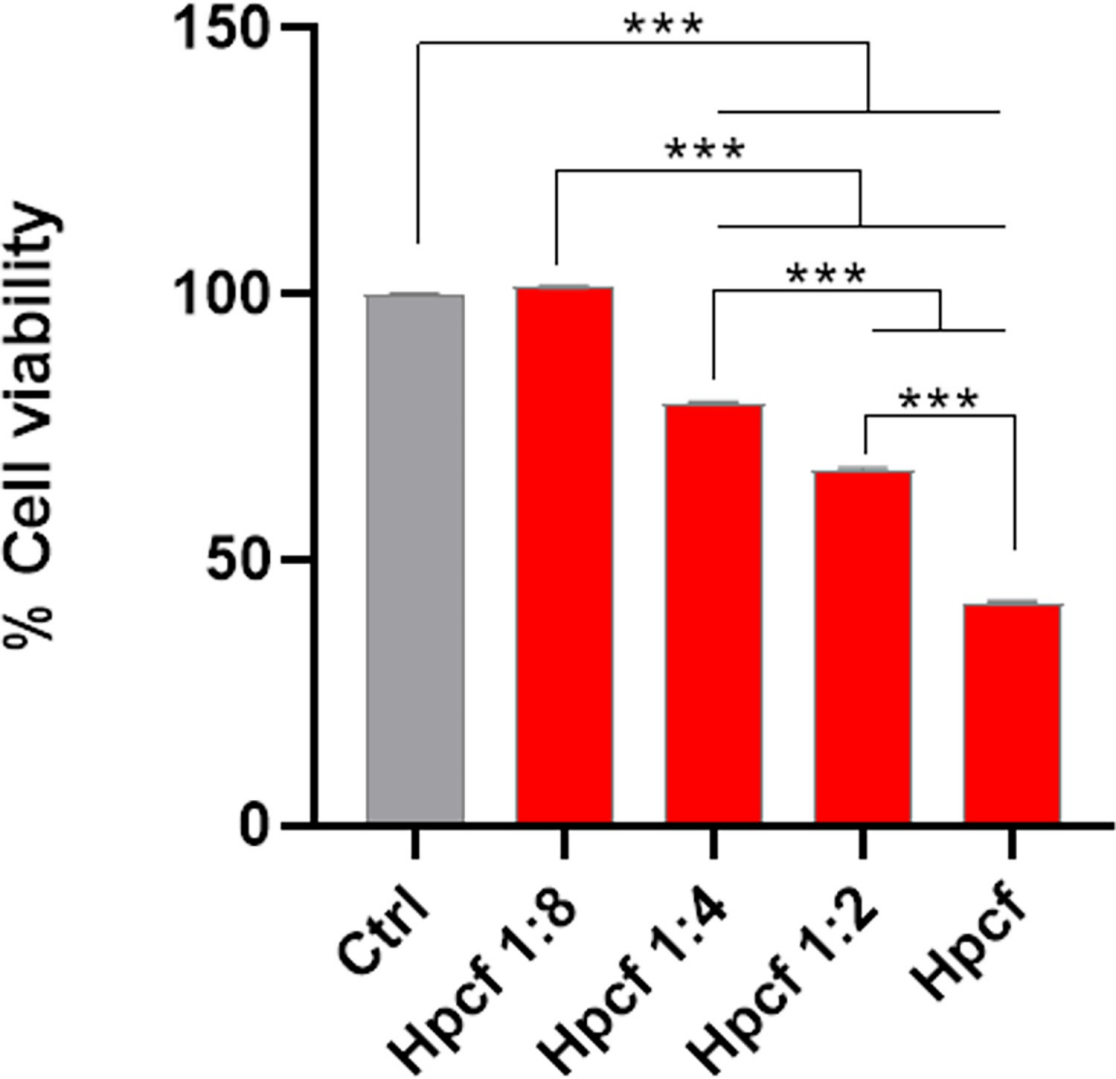
Hpcf affects gastric epithelial cell viability. MTT assay showing the AGS cells viability after incubation with different concentrations of Hpcf for 24 hours. The assay was performed testing Hpcf as it is (Hpcf) or diluted with uninoculated broth medium (Hpcf 1:2; Hpcf 1:4; Hpcf 1:8). One-way ANOVA followed by Bonferroni post hoc correction was used to determinate statistically significant differences (*** p <0.0001).

It has been reported that the *H*. *pylori* culture filtrate (Hpcf) from VacA^+^ P12 strain inhibits AGS cell proliferation, inducing cell death by apoptosis [17]. In agreement with this finding, AGS cells stimulated with Hpcf from P12 strain showed a statistically significant viability decrease, compared to control cells (Fig 6). Notably, this result was found to be dependent on the Hpcf concentration. In fact, when eightfold diluted, Hpcf did not display cytotoxic effect (Fig 6), since cell viability was close to that of control cells.

**Fig 7:**
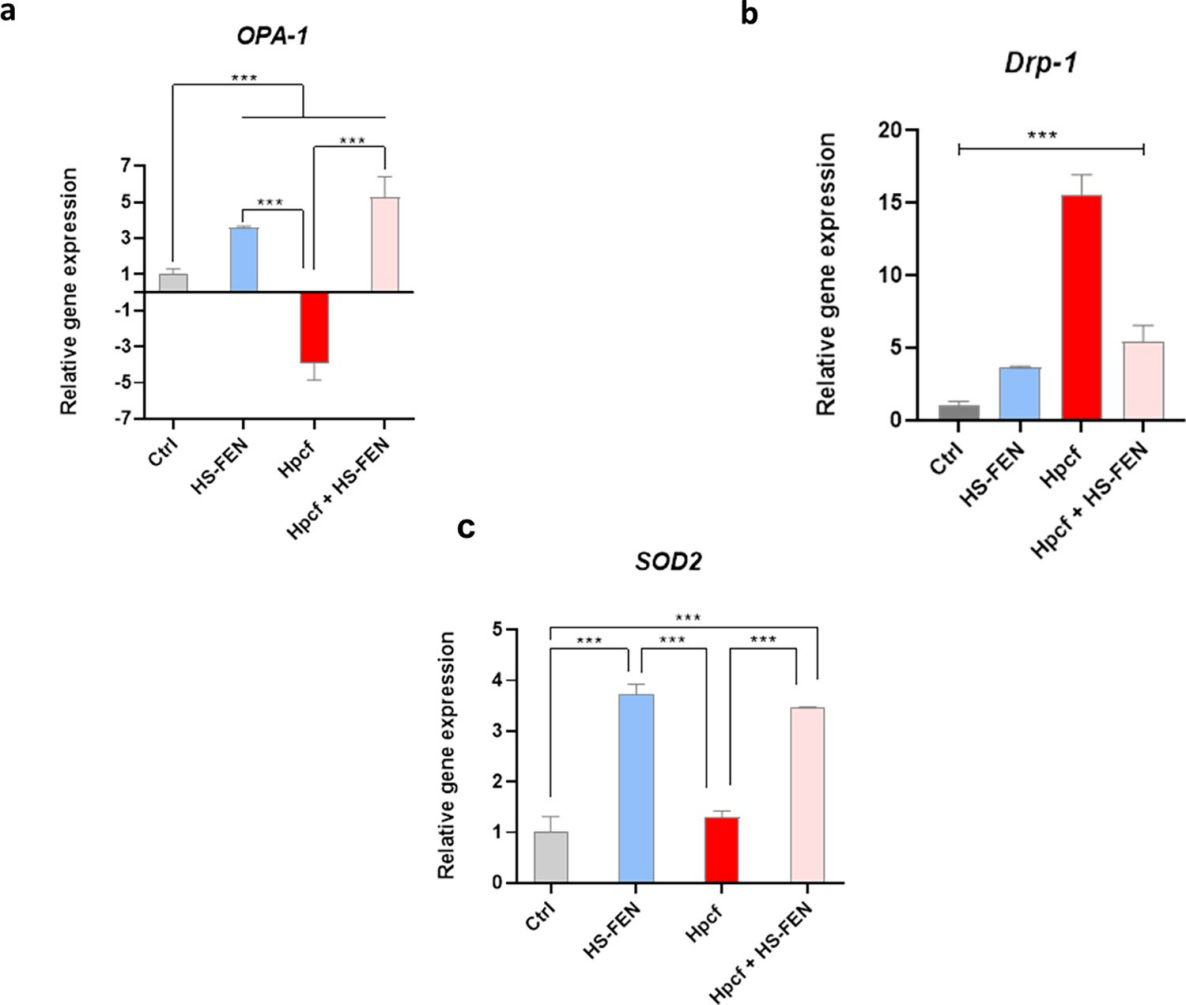
HS-FEN subverts detrimental effects elicited by Hpcf. Cellular expression of *OPA-1* (**a**), *Drp-1* (**b**) and *SOD2* (**c**) genes detected by quantitative PCR performed on RNA extracted from AGS cells cultured for 3 hours with HS-FEN 25 μg mL^-1^ in presence or absence of Hpcf (1:2) stimulation. *GAPDH* was used as housekeeping gene to normalize all samples. Data were represented as means ± s.d. of three independent experiments, each performed in triplicate. One-way ANOVA followed by Bonferroni post hoc correction was used to determinate statistically significant differences (*** p <0.0001).

The pro-apoptotic activity of Hpcf was confirmed by analyzing the gene expression of both *OPA-1* and *Drp-1* genes. Results revealed a significant decrease of *OPA-1* gene expression and increase of *Drp-1* gene expression after stimulation with concentrated Hpcf (Fig 7A and 7B). OPA-1, along with Drp-1 play critical roles in preserving mitochondrial morphology and function. They are recognized as “mitochondria-shaping” proteins, responsible for mitochondria dynamic regulation through a careful balance between fission and fusion events [55]. Down-regulation of OPA-1, together with up-regulation of Drp-1, favor mitochondria fragmentation and cristae remodeling, facilitating the release of cytochrome c triggered by apoptotic stimuli [56,57]. Therefore, our results suggest that Hpcf affects mitochondrial functions, favoring the process of mitochondrial fission.

Mitochondria are the main source of reactive oxygen species (ROS). Alteration of mitochondria, promoted by loss of OPA-1 and/or Drp-1 accumulation, leads to excessive ROS production [57–59]. The evidence of the reduced expression of *SOD2* gene in cells incubated with Hpcf (Fig 7B), represents a further proof confirming the deleterious role of Hpcf by causing mitochondrial dysfunction and therefore, apoptosis and oxidative stress [60].

Interestingly, HS-FEN treatment was found to increase the expression of both *OPA-1* and *SOD2* genes in cells stimulated or not with Hpcf (Fig 7A and 7C). On the contrary, HS-FEN treatment was found to decrease the expression of *Drp-1* gene in cells stimulated or not with Hpcf, compared to Hpcf-stimulated cells (Fig 7B). Remarkably, cells treated with HS-FEN alone also expressed high levels of *Drp-1* gene, compared to untreated cells. However, the increased levels of *Drp-1*, following HS-FEN treatment, were not remarkable compared to Hpcf-stimulated cells (Fig 7B). This result may seem a contradiction, but it may find explanation in the involvement of Drp-1 –as mitochondrial fission protein–also in cell proliferation and mitosis [61].

These results suggest: 1) the protective role of HS-FEN under basal conditions and 2) the beneficial effect of HS-FEN during *H*. *pylori* infection, by limiting mitochondrial ROS production and preserving cells from mitochondrial dysfunction.

Recently, it has been demonstrated that *OPA-1* silencing also causes IkB degradation and NF-kB activation, promoting the expression of pro-inflammatory genes [62,63]. Consequently, OPA-1 plays key roles in the NF-kB pathway.

**Fig 8:**
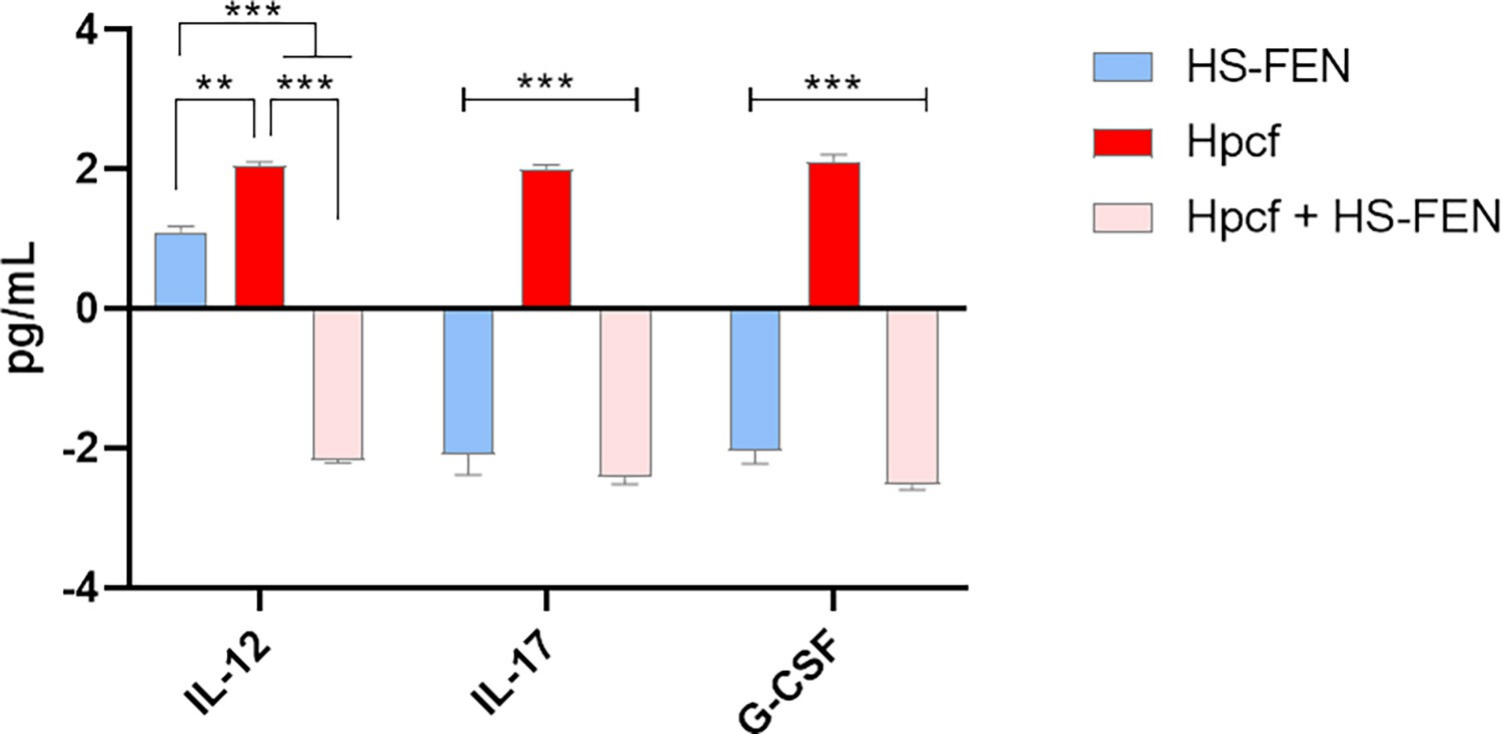
HS-FEN mitigates Hpcf-induced inflammation. Expression level of cytokines IL-12, IL-17 and G-CSF detected in AGS culture medium. Graph reports pg of cytokine in mL of cell medium differently treated: 1) HS-FEN 25 μg mL^-1^; 2) Hpcf 1:2 for 12 hours; 3) Hpcf 1:2 + HS-FEN 25 μg mL^-1^ for 12 hours. Values were normalized to basal activity (control cells) and data were represented as means ± s.d. of three independent experiments, each performed in triplicate. One-way ANOVA followed by Bonferroni post hoc correction was used to determinate statistically significant differences (** p<0.001; *** p <0.0001).

In line with this finding, our results showed increased expression of IL-12, IL-17 and G-CSF cytokines in Hpcf stimulated cells. IL-17 is a pro-inflammatory cytokine playing critical roles in the pathogenesis of chronic inflammatory diseases. It is produced during the *H*. *pylori* infection and contributes to gastric mucosa damage, by inducing pro-inflammatory mediators such as the granulocyte colony-stimulating factor (G-CSF), which participates to the acute phase of inflammation [64,65]. IL-12, in turn, is a pro-inflammatory cytokine released during infections and functions as point of connection between the innate and adaptative immunity [66]. As expected, HS-FEN treatment was found to decrease the release of IL-12, IL-17 and G-CSF by Hpcf (Fig 8).

Due to the complex molecular heterogeneity of HS-FEN, as demonstrated by both NMR and IR spectra (Figs 1 and 2), molecular basis of protective and beneficial effects exhibited by this humic matter cannot be easily predicted. It seems plausible to attribute the protective effect of HS-FEN and, more generally, of humic substances to their reducing properties (Fig 4). As shown by spectroscopic results, in fact, HS-FEN is rich in phenolic and lignin components (Tables 2 and 3) which possess important antioxidant properties, by scavenging ROS [45,47]. Consequently, HS-FEN attenuates the impact of the oxidative stress on mitochondrial dynamics and mitigates the inflammatory process protecting the cells from the oxidative stress [50].

However, a clear relationship between chemical structure and anti-inflammatory features of humic substances has not yet clearly elucidated. A decreased release of IL-12, IL-17 and G-CSF by HS- FEN treatment could find explanation in the conformational and structural properties of humic molecules and the large content of polyphenolic or lignin components (Tables 1 and 2, Fig 2). Notably, the most reasonable explanation about the capability of HS-FEN to suppress the expression of the Hpcf-induced cytokines could reside in the hydrophobic feature and conformational flexibility of this substance. The combined characteristic can promote a surface adhesion to cell membranes and a subsequent disruption of the humic suprastructures into smaller humic associations, from which, by interacting with eukaryotic cells, small bioactive phenolic molecules may be concomitantly released, and determine a significant reduction of the inflammatory response.

### Limitations of study

Nevertheless, some limitations of the study must be noted. The major advantages of using products of recycled agricultural biomasses—such as green composts—in pharmacological therapy, consist in sustainability and renewability, as well as large availability of bioactive molecules. Among the various humic components, we attributed the antioxidant and anti-inflammatory role of HS-FEN to its abundant phenolic and lignin content, hypothesizing their synergistic action, but we did not determine the individual effects of these components in mitigating the inflammatory response. This topic remains one of the main goals of our next studies.

Moreover, apart from a clearer determination of the HS-FEN composition which may facilitate the assignment of its biological properties to individual chemical components, the potential use of HS-FEN as therapeutic drug also requires more pharmacological evidences. In particular, the toxicological safety of HS-FEN has to be assessed, in order to uncover probable clinical adverse effects.

Lastly, additional *in vivo* studies are needed to confirm the efficacy of HS-FEN, in order to verify the potentiality of humic substances from composted green biomasses, not only in agricultural sector as biostimulants [67], but also in medical field.

## Conclusion

This work showed a key role of HS-FEN in favoring mitochondrial homeostasis, modulating both the inflammation and the oxidative stress. Mitochondria are considered “metabolic checkpoints” able to sense the metabolic status of cells. It is not surprising that when metabolic changes–due to invading pathogens and/or inflammatory events–occurs, mitochondria alter their functions. Thus, mitochondrial dysfunction reflects the cellular metabolic status and may result in both oxidative stress and alteration of fission and fusion proteins [68]. In the present study, factors critical for mitochondrial dynamics were found dysregulated upon Hpcf stimulation. *OPA-1* and *SOD-2* genes decreased in Hpcf-stimulated cells, while *Drp-1* gene increased, suggesting the contribution of Hpcf in mitochondria fragmentation, which in turn contributed to the unlimited production of reactive oxygen species and cell apoptosis. In addition, mitochondrial fragmentation, via OPA-1 dysregulation, also promotes the NF-kB activation and consequent inflammation [63]. This finding was confirmed, in this study, by up-regulation of IL-17, IL-12 and G-CSF cytokines. All these alterations were reverted following HS-FEN treatment. Therefore, HS-FEN seems to restore normal mitochondrial functions and attenuate the *H*. *pylori*-associate inflammatory response. These effects may be attributed to: 1) the considerable content of phenolic and lignin components in HS-FEN, 2) its hydrophobic components, and, 3) the flexibility of its supramolecular conformation. Given this scenario, HS-FEN could represent a valid alternative against the *H*. *pylori*-associated inflammation and related disorders.

## Supporting information

S1 Fig Total ion chromatograms of thermochemolysis products of HS-FEN. (TIF)

S1 Table List of the main products released by the thermochemolysis from humic substances from fennel (HS-FEN). (PDF)

S2 Table Primers used for qRT-PCR. (PDF)

S1 Dataset (DOCX)
