# Supplementary figures and images for: Humic substances from composted fennel residues control the inflammation induced by Helicobacter pylori infection in AGS cells
Source: PLoS One. 2023 Mar 9;18(3):e0281631. doi: 10.1371/journal.pone.0281631 (PMC9997894; doi:10.1371/journal.pone.0281631)

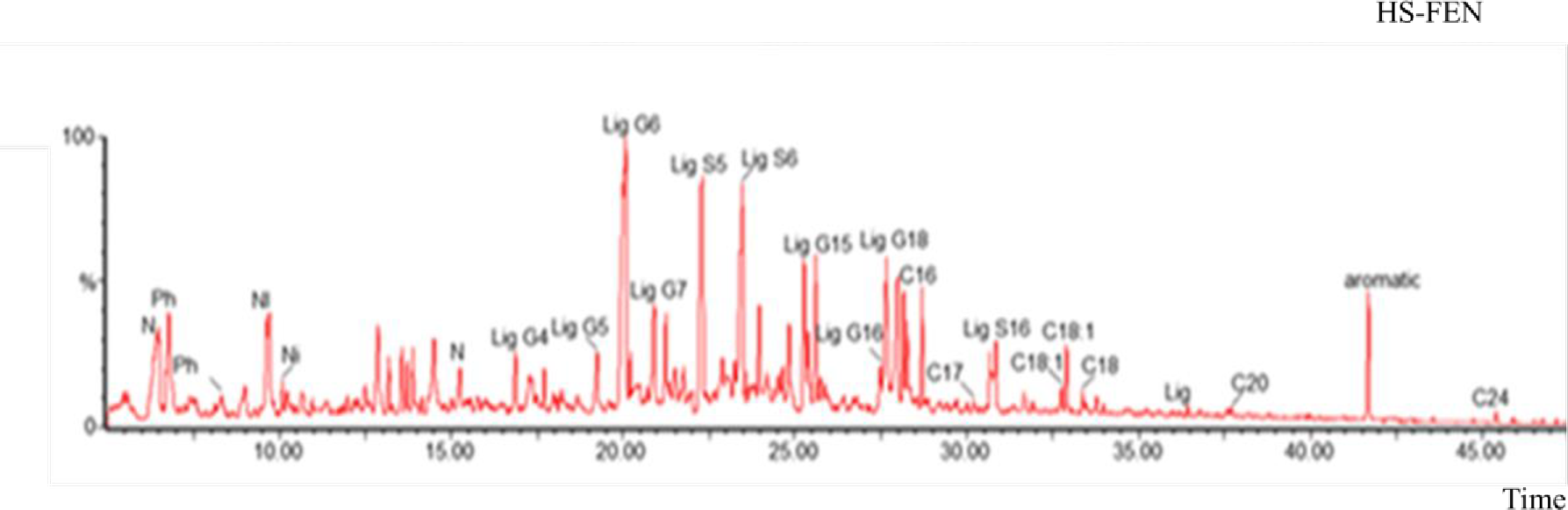

Supplement: S1 Fig — (TIF) [file pone.0281631.s001.tif]
