# Supplementary material for: Humic substances from composted fennel residues control the inflammation induced by Helicobacter pylori infection in AGS cells
Source: PLoS One. 2023 Mar 9;18(3):e0281631. doi: 10.1371/journal.pone.0281631 (PMC9997894; doi:10.1371/journal.pone.0281631)
Supplement: S1 Table — (PDF) [file pone.0281631.s002.pdf]

HS-FEN

RT[a]	Compound	Origin[b]
7.01	phenol	phenol
7.24	methoxy benzene	P1 Lig
8.4	1-ethenyl-4 CH3O-benzene	P3 Lig
8.61	1,2-di-CH3O benzene	G1 Lig
8.95	dimethoxy benzene	carbohydrate
9.74	m/z 128	N
9.88	N derivate	N
10.03	N derivate	N
10.39	3,4-dimethoxy toluene	G2 Lig
10.7	2,6-dimethoxy phenol	carbohydrate
11.07	Benzaldehyde methoxy	P4 Lig
11.28	1-methyl-1H-indole	N
12.31	1,2,3-tri-CH3O benzene	S1 Lig
12.56	1-(4-methoxyphenolonyl)-ethanonel)	P5 Lig
12.87	m/z 128	N
13.23	m/z 98	N
13.56	dimethoxy phenol	carbohydrate
13.9	4-ethenyl-1,2-dimethoxy-benzene	G3 Lig
14.02	1,2,4-trimethoxy benzene	carbohydrate
14.2	benzoic acid 4-methoxy methyl ester	P6 Lig
14.49	2-Propenoic acid, 3-phenolonyl methyl ester (e)	lipid
14.8	1,2,3-methoxy 5-methyl benzene	S2 Lig
15.35	m/z 128	N
15.61	m/z 128	N
15.95	1,3-diidro-1-metil-2H-indol-2-one	N
16.1	5-metossi-2-metil-1H-indole	N
16.37	N derivative	N
17.25	3,4-dimethoxy-benzaldehyde	G4 Lig
17.59	1,2-dimethoxy-4-(1-propenyl) benzene	G21 Lig
18	2,5-dimethoxy-N,N-dimethylbenzylamine	G7 -G8 Lig
18.49	benzenepropanoic acid, 4-methoxy, methyl ester	P12 Lig
18.58	1,2,3,4-tetramethoxy benzene	carbohydrate
19.59	1-(3,4-dimethoxyphenolonyl)-ethanone	G5 Lig
20.35	3,4-dimethoxy-methyl ester benzoic acid	G6 Lig
20.53	3,4,5-trimethoxy benzaldehyde	S4 Lig
21	m/z 98	N
21.22	cis-1-methoxy-2-(3,4-dimethoxyphenolonyl) ethylene	G7 Lig
21.56	trans-1-methoxy-2-(3,4-dimethoxyphenolonyl) ethylene	G8 Lig
21.77	trans-1-methoxy-1-(3,4-dimethoxyphenolonyl)-1-propene	G11 Lig
22.61	2-propenoic acid, 3-(4-methoxyphenolonyl)-methyl ester	P18 Lig
22.61	1-(3,4,5-trimethoxyphenolonyl)-ethanone	S5 Lig
23.76	benzoic acid, 3,4,5-trimethoxy-methyl ester	S6 Lig
24.25	trans-3-methoxy-1-(3,4-dimethoxyphenolonyl)-1-propene	G13 Lig
25.14	cis-1-(3,4,5-trimethoxyphenolonyl)-2-methoxy ethylene	S7 Lig
25.43	C15 iso FAME (C14, 13-methyl, FAME)	mic
25.49	1-(3,4-di-CH3O phenolonyl)-1,2,3-tri CH3O propane (three/erythro)	G14 Lig

25.64	C15 ante iso FAME (C14, 12 methyl, FAME)	mic
25.64	trans-1-(3,4,5-trimethoxyphenolonyl)-2-methyl ethylene	S8 Lig
25.84	1,2,3-trimethoxy-1-(3,4-dimethoxyphenolonyl) propane (threetoheritro)	G15 mic
26.13	cis-1-(3,4,5-trimethoxyphenolonyl)-1-methoxy-1-propene	S10 Lig
27.76	cis-1-(3,4,5-trimethoxyphenolonyl)-1,3-dimethoxy-1-propene	G16 Lig
27.98	propenoic acid trans-3-(3,4-di-CH3O phenolonyl)-3CH3O	G18 Lig
28.21	1,2,3-trimethoxy-1-(3,4,5-trimethoxyphenolonyl) propane (three/peritre)	S14 Lig
28.32	C16:1 FAME	mic
28.48	1,2,3-trimethoxy-1-(3,4,5-trimethoxyphenolonyl) propane (three parts per liter)	S15 Lig
28.88	C16 FAME	lipid
28.98	cis-1-(3,4,5-trimethoxyphenolonyl)-1,3-dimethoxy-prop-1-ene	S16 Lig
30.29	trans-2-propenoic acid-3-(3,4,5-trimethoxyphenolinyl)-methyl ester	S18 Lig
30.37	C17 FAME iso (C16, 15 methyl, FAME)	mic
30.56	C17 ante iso FAME (C16, 14 methyl, FAME)	mic
31.1	cis-1-(3,4,5-trimethoxyphenolonyl)-1,3-dimethoxy-prop-1-ene	S16gLig
32.9	C18:1 FAME	lipid
33.04	C18:1 FAME	lipid
33.53	C18 FAME	lipid
33.76	podocarp-7-en 3-one dimethyl isomer	lipid
34.02	podocarp-7-en 3-one dimethyl isomer	lipid
36.69	sterol	sterol
37.82	C20 FAME	lipid
37.98	N derivate	N
39.16	N derivate	N
39.92	N derivate	N
40.05	sterol	sterol
41.79	C22 FAME	lipid
43.66	C23 FAME	lipid
45	3,3',4,4'-tetramethoxystilbene	aromatic
45.48	C24 FAME	lipid
46.84	squalene	sterol
47.22	C26-Ome	alcohol
47.6	aromatic	aromatic
48.38	alkane	alkane
48.93	C26 FAME	lipid
49.43	C24, 24-CH3O, FAME	lipid
50.54	C28-CH3O	alcohol
52.17	C28 FAME	lipid
54.42	sterol	sterol
55.25	C30 FAME	lipid

[a] RT retention time (min)
[b] FAME fatty acid methyl ester; Lg lignin: P phydroxyphenyl, G guayacil, S syringyl; Lip: plant lipids; ME methyl ester; Mic microbial origin; N: nitrogen derivative
