# Supplementary material for: Humic substances from composted fennel residues control the inflammation induced by Helicobacter pylori infection in AGS cells
Source: PLoS One. 2023 Mar 9;18(3):e0281631. doi: 10.1371/journal.pone.0281631 (PMC9997894; doi:10.1371/journal.pone.0281631)
Supplement: S2 Table — (PDF) [file pone.0281631.s003.pdf]

GENE	FORWARD PRIMER	REVERSE PRIMER
GAPDH (Glyceraldehyde 3-Phosphate Dehydrogenase)	CCTCTGACTTCAACAGCGACAC	CACCACCCTGTTGCTGTAGCCA
SOD2 (Superoxide Dismutase 2)	CTGATTTGGACAAGCAGCAA	CTGGACAAACCTCAGCCCTA
OPA1 (Optic Atrophy Protein 1)	GCCGGAAGTGTAGTTACCTG	AGGTGGTCTCTGTGGGTTGT
Drp1 (Dynamin Related Protein 1)	GATGCCATAGTTGAAGTGGTGAC	CCACAAGCATCAGCAAAGTCTGG
