# Supplementary material for: Humic substances from composted fennel residues control the inflammation induced by Helicobacter pylori infection in AGS cells
Source: PLoS One. 2023 Mar 9;18(3):e0281631. doi: 10.1371/journal.pone.0281631 (PMC9997894; doi:10.1371/journal.pone.0281631)
Supplement: S1 Dataset — (DOCX) [file pone.0281631.s004.docx]

**MINIMAL DATA SET**

**Cell viability assay following HS-FEN incubation**

|  | **Sample 1** | **Sample 2** | **Sample 3** | **Avg** | **Standard deviation** | **% Cell viability** |
| --- | --- | --- | --- | --- | --- | --- |
| **Ctrl** | 0,729 | 0,746 | 0,914 | 0,796333 | 0,1022562 |  |
| **HS-FEN 500 μg/mL** | 0,4 | 0,444 | 0,404 | 0,416 | 0,0243311 | 33,73986 |
| **HS-FEN 250 μg/mL** | 0,622 | 0,525 | 0,51 | 0,552333 | 0,0607975 | 57,49132 |
| **HS-FEN 100 μg/mL** | 0,491 | 0,618 | 0,601 | 0,57 | 0,068942 | 60,56914 |
| **HS-FEN 50 μg/mL** | 0,637 | 0,744 | 0,648 | 0,676333 | 0,0588586 | 79,09412 |
| **HS-FEN 25 μg/mL** | 0,743 | 0,714 | 0,893 | 0,816667 | 0,0750355 | 97,7352 |
| **HS-FEN 12.5 μg/mL** | 0,85 | 0,987 | 0,759 | 0,865333 | 0,1147708 | 112,021 |
| **HS-FEN 6 μg/mL** | 0,953 | 0,948 | 0,758 | 0,886333 | 0,111168 | 115,6795 |
| **BLANK** | 0,246 | 0,179 | 0,242 | 0,222333 | 0,037581 |  |

Data were represented as Log(inhibitor) vs normalized response.

**Cell viability assay following Hpcf incubation**

|  | **Sample 1** | **Sample 2** | **Sample 3** | **Average** | **Standard deviation** | **% Cell viability** |
| --- | --- | --- | --- | --- | --- | --- |
| **Ctrl** | 0.373 | 0.394 | 0.371 | 0.379333 | 0.01274101 |  |
| **Hpcf 1:8** | 0.413 | 0.363 | 0.377 | 0.384333 | 0.035355339 | 101.5151515 |
| **Hpcf 1:4** | 0.402 | 0.309 | 0.235 | 0.315333 | 0.083679946 | 80.60606061 |
| **Hpcf 1:2** | 0.243 | 0.302 | 0.275 | 0.273333 | 0.02953529 | 67.87878788 |
| **Hpcf** | 0.217 | 0.182 | 0.171 | 0.19 | 0.024020824 | 42.62626263 |
| **BLANK** | 0.049 | 0.048 | 0.051 | 0.049333 |  |  |

**Antioxidant activity of HS-FEN by ABTS Assay**

| **HS-FEN μg/mL** | **% Inhibition** |
| --- | --- |
| 50 | 50.868 |
| 30 | 30.567 |
| 25 | 19.722 |
| 0 | 0.500 |

***OPA-1, Drp1* and *SOD2* genes expression**

| ***OPA-1*** | **Fold Regulation** | | |  |  |
| --- | --- | --- | --- | --- | --- |
|  | **Sample 1** | **Sample 2** | **Sample 3** | **Average** | **Standard deviation** |
| **Hpcf** | -4.74 | -3.89 | -2.91 | -3.846666667 | 0.915769258 |
| **HS-FEN** | 3.6 | 3.55 | 3.63 | 3.593333333 | 0.040414519 |
| **Hpcf + HS-FEN** | 7.01 | 5.361 | 5.5 | 5.957 | 0.914569298 |

| ***Drp1*** | **Fold Regulation** | | |  |  |
| --- | --- | --- | --- | --- | --- |
|  | **Sample 1** | **Sample 2** | **Sample 3** | **Average** | **Standard deviation** |
| **Hpcf** | 1.43 | 1.24 | 1.15 | 1.273333333 | 0.142945211 |
| **HS-FEN** | 3.59 | 4.31 | 3.23 | 3.71 | 0.549909083 |
| **Hpcf + HS-FEN** | 3.47 | 3.44 | 3.45 | 3.453333333 | 0.015275252 |

| ***SOD-2*** | **Fold Regulation** | | |  |  |
| --- | --- | --- | --- | --- | --- |
|  | **Sample 1** | **Sample 2** | **Sample 3** | **Average** | **Standard deviation** |
| **Hpcf** | 17.32 | 14.44 | 15.92 | 15.89333333 | 1.440185173 |
| **HS-FEN** | 3.74 | 3.67 | 3.48 | 3.63 | 0.13453624 |
| **Hpcf + HS-FEN** | 6.03 | 4.01 | 6.39 | 5.476666667 | 1.282861385 |

**Multiplex for cytokines measurement**

| **IL-12** | **Obs. Conc. compared to control group** | | |  |  |
| --- | --- | --- | --- | --- | --- |
|  | **Sample 1** | **Sample 2** | **Sample 3** | **Average** | **Standard deviation** |
| **Hpcf** | 2.078 | 2.13 | 2.03 | 2.079333 | 0.050013332 |
| **HS-FEN** | 1.27 | 1.11 | 1.01 | 1.13 | 0.13114877 |
| **Hpcf + HS-FEN** | -2.15 | -2.18 | -2.13 | -2.15333 | 0.025166115 |

| **IL-17** | **Obs. Conc. compared to control group** | | |  |  |
| --- | --- | --- | --- | --- | --- |
|  | **Sample 1** | **Sample 2** | **Sample 3** | **Average** | **Standard deviation** |
| **Hpcf** | 1.95 | 2.04 | 2.01 | 1.995 | 0.06363961 |
| **HS-FEN** | -2.45 | -2.02 | -1.94 | -2.13667 | 0.274286954 |
| **Hpcf + HS-FEN** | -2.54 | -2.49 | -2.42 | -2.48333 | 0.060277138 |

| **G-CSF** | **Obs. Conc. compared to control group** | | |  |  |
| --- | --- | --- | --- | --- | --- |
|  | **Sample 1** | **Sample 2** | **Sample 3** | **Average** | **Standard deviation** |
| **Hpcf** | 2.15 | 2.13 | 2.08 | 2.12 | 0.036055513 |
| **HS-FEN** | -2.13 | -1.97 | -2.07 | -2.05667 | 0.080829038 |
| **Hpcf + HS-FEN** | -2.57 | -2.51 | -2.48 | -2.52 | 0.045825757 |
